# Perioperative management of patients with primary aldosteronism: a practical approach for the endocrinologist

**DOI:** 10.1530/EC-26-0178

**Published:** 2026-06-18

**Authors:** Patrícia da Cunha Brito, Adriana de Sousa Lages, Catarina Machado, Vera Fernandes

**Affiliations:** ^1^ Department of Endocrinology, Unidade Local de Saúde de Braga, Braga, Portugal; ^2^ School of Medicine, University of Minho, Braga, Portugal; ^3^ Faculty of Medicine, University of Coimbra, Coimbra, Portugal

**Keywords:** primary aldosteronism, perioperative management, adrenalectomy, endocrine hypertension

## Abstract

Primary aldosteronism (PA) is the most common endocrine cause of secondary hypertension yet remains significantly underdiagnosed. Early recognition is essential, as excess aldosterone leads to substantial cardiovascular and renal morbidity. Approximately one-third of patients have unilateral disease, in whom adrenalectomy is associated with superior long-term clinical outcomes compared with medical therapy. Although current guidelines offer detailed recommendations for diagnosis and subtype classification, guidance on perioperative management remains limited. Preoperative goals should include optimizing blood pressure to <140/90 mmHg and correcting potassium levels, which can be achieved by initiating mineralocorticoid receptor antagonists (MRAs) in all patients. MRA titration ensures effective receptor blockade and supports recovery of contralateral adrenal function. A dedicated preoperative endocrinology consultation is essential to review expected surgical outcomes, address the anticipated postoperative decline in renal function, evaluate cortisol co-secretion, and assess the risk of postoperative adrenal insufficiency. Postoperatively, the primary concern is hyporeninemic hypoaldosteronism. Antihypertensive medications should be down-titrated, MRAs withheld, and a high-sodium diet encouraged. Early biochemical assessment does not establish cure but may still provide clinically relevant information. Follow-up includes weekly assessments during the first month and structured evaluation of clinical and biochemical outcomes at 6–12 months using the PASO criteria. Histological findings help predict recurrence risk and decide long-term follow-up. Patients achieving complete biochemical success, along with a complete or partial clinical response and having classic histopathological features, may transition to long-term follow-up in a primary care setting. This manuscript proposes a structured perioperative management approach for patients with PA undergoing adrenalectomy.

## Introduction

Primary aldosteronism (PA) is the leading endocrine cause of secondary hypertension (HT), with a prevalence estimated at 5–14% among all hypertensive patients and up to 30% in specialized referral centers (1, 2). Despite its high frequency, PA remains significantly underdiagnosed, largely due to the misconception that diagnostic algorithms are overly complex.

Early recognition is essential, as excessive levels of aldosterone contribute not only to HT but also to substantial end-organ damage, particularly involving the cardiovascular and renal systems (1, 3, 4, 5, 6, 7). Patients with PA exhibit significantly greater cardiovascular morbidity and mortality compared with age-, sex-, and blood pressure (BP)-matched individuals with essential HT (7, 8, 9, 10). Moreover, PA patients also report a diminished quality of life (1, 7, 11, 12). For these reasons, treatment goals must extend beyond normalization of BP and include management of aldosterone excess, achieved either through adrenalectomy, which corrects hormonal overproduction, or through mineralocorticoid receptor antagonism, which blocks aldosterone action (1, 3, 4, 7, 13).

Approximately one-third of PA cases result from unilateral disease, most often from an aldosterone-producing adenoma and less frequently from unilateral hyperplasia (3, 7, 13, 14). When feasible, unilateral total adrenalectomy remains the preferred treatment, achieving normalization of HT in 30–60% of patients and improving BP control in the majority of cases (3, 4, 5, 7, 15). Hypokalemia typically resolves, and patients report improved quality of life compared with those receiving medical therapy (1, 4, 5, 7). Furthermore, surgery has been associated with better long-term BP control, requiring fewer antihypertensive medications at lower doses, as well as reduced cardiovascular risk and mortality (1, 2, 4, 5, 15, 16, 17).

**Figure 1:**
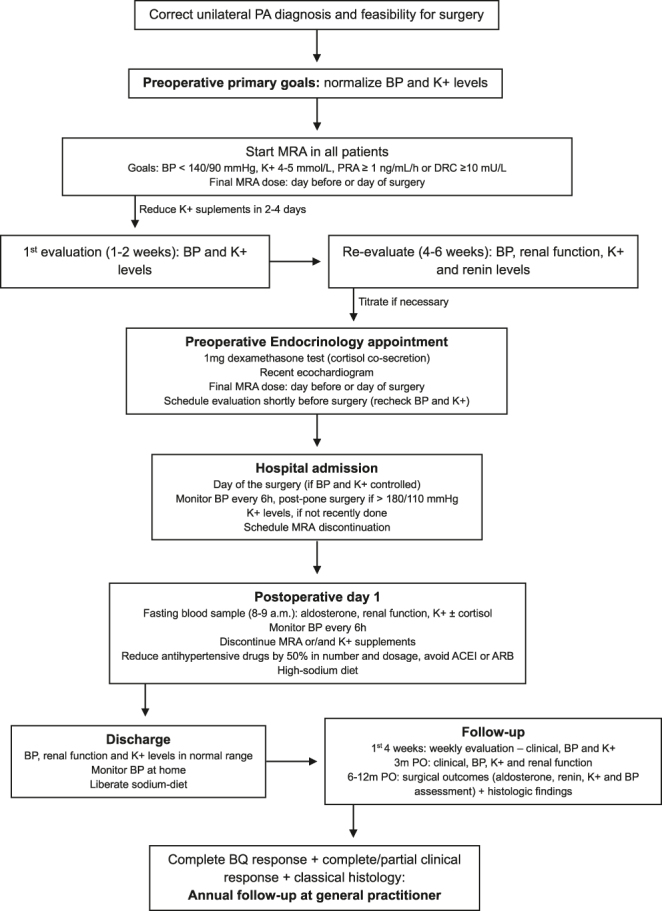
Perioperative management algorithm.

However, while current guidelines offer comprehensive recommendations on diagnosis and subtype classification, they provide limited discussion regarding specific perioperative management, despite its clinical importance. Thoughtful management before, during, and after adrenalectomy contributes to better outcomes and facilitates clinical decision-making. This manuscript presents a comprehensive perioperative approach, outlining the rationale for key management decisions and integrating relevant published recommendations. A structured perioperative algorithm forms the core of the manuscript (Fig. 1), illustrating the proposed management strategy for each stage of care, including preoperative preparation, hospital admission, and postoperative management.

## Preoperative management

The preoperative phase represents the first step of the proposed perioperative algorithm and focuses on accurate subtype classification, BP optimization, and correction of potassium disturbances.

Establishing unilateral disease is the cornerstone of preoperative workup, as accurate lateralization directly influences treatment outcomes (13, 18). Adrenal venous sampling (AVS) performed in experienced centers is essential, as imaging modalities such as CT or MRI alone cannot assess adrenal functionality and are therefore insufficient for subtype differentiation (1, 4, 5, 7, 18). Although a detailed discussion of AVS is beyond the scope of this manuscript, it is important to emphasize that bilateral adrenal abnormalities on CT do not necessarily indicate bilateral PA, and, conversely, the presence of a solitary adrenal nodule does not confirm unilateral disease. An exception applies to patients younger than 35 years who present with severe PA and a unilateral adrenal mass larger than 1 cm, in whom AVS can be safely bypassed and surgery pursued directly (1).

### Primary goals and mineralocorticoid receptor antagonist (MRA) therapy

The primary goals of preoperative preparation are to optimize BP and potassium levels (2, 4, 5, 7, 13). Although there is no current evidence defining optimal BP targets before surgery or their impact on outcomes, BP is generally recommended to be controlled to values below 140/90 mmHg, and surgery should be postponed when BP exceeds 180/110 mmHg, in accordance with published recommendations (5, 13, 19, 20). BP should be monitored routinely to ensure adequate control. To facilitate the subsequent assessment of surgical outcomes, physicians should request ambulatory documentation of BP levels, as well as a complete record of all antihypertensive medications and their dosages, for future comparison (6). When home BP surveillance is not feasible, 24 h ambulatory BP monitoring may be used as an alternative.

An MRA should be initiated in all patients without contraindications, ideally at least four weeks prior to surgery (5, 13, 19, 21). This strategy provides both short- and long-term benefits. In the short term, MRAs can improve BP control and normalize potassium levels, thereby reducing the risk of intraoperative complications such as arrhythmia and hemodynamic instability (22, 23, 24). From a long-term perspective, MRAs help prevent further end-organ damage while patients await surgery and may facilitate the functional recovery of the contralateral adrenal gland (1, 2). Furthermore, Goi *et al.* reported that MRA use was not associated with an increased risk of hyperkalemia, deterioration in renal function, or excessive decreases in BP in the early postoperative period. Although robust evidence for preoperative benefit of MRAs is limited, their use is supported by physiological rationale. Therefore, they should still be considered, even in patients with previously normal potassium levels and/or well-controlled BP. MRA therapy should be titrated to achieve BP below 140/90 mmHg, potassium levels between 4 and 5 mmol/L, and direct renin concentration greater than 10 mU/L or plasma renin activity above 1 ng/mL/h. These targets reflect effective mineralocorticoid receptor blockade (1, 25, 26).

Spironolactone remains the first-line MRA due to its low cost, wide availability, and proven efficacy (1, 3, 4, 13, 27). However, as a non-selective MRA, it is associated with dose-dependent adverse effects: in males, its antiandrogenic activity can lead to gynecomastia and erectile dysfunction, while in females, its progestogenic effects may cause menstrual irregularities (4, 5, 7, 15, 27). Eplerenone is a selective MRA and therefore does not cause the previously mentioned endocrine side effects (4, 13). Its use in PA is off-label in several countries, as regulatory approval is limited to heart failure indications (28). Eplerenone is approximately 25–50% less potent than spironolactone on a milligram-to-milligram comparison, such that higher doses will be required (4, 7, 13, 15). Finerenone has emerged as a potential therapeutic option, although evidence supporting its use in PA is limited and remains off-label (29). Currently, finerenone is only approved by the European Medicines Agency for chronic kidney disease (CKD) with albuminuria associated with type 2 diabetes (30). A small real-world study reported poorer biochemical and clinical responses when compared with eplerenone (31). Nevertheless, these findings may reflect inadequate titration of finerenone to doses equivalent to those of eplerenone. Additionally, the Endocrine Society’s 2025 guidelines recommend administering finerenone once daily (1). However, given its relatively short half-life of 2–3 h, it is reasonable to question whether a twice-daily regimen may be more appropriate (32). Table 1 presents the essential properties of MRAs and practical guidance for their use (1, 2, 3, 4, 7, 13, 15, 27, 33). It should be noted that all MRAs are expected to offer similar efficacy in mineralocorticoid receptor blockade when titrated to pharmacologically equivalent doses (1, 15).

**Table 1:** MRAs for treatment of PA.

	Spironolactone	Eplerenone	Finerenone
Selectivity	Non-selective	Selective	Selective
Initial dose	12.5–25 mg/daily	25–50 mg/twice daily	Unknown
			10–20 mg
Conditions	Once daily	Twice daily	Unknown
	With food	With or without food	With or without food
Dose increments	25–50 mg	25–100 mg	Unknown
Maximum dose	200–400 mg/daily*	200–300 mg/twice daily^†^	Unknown
Available formulations in Portugal	25 mg	25 mg	10 mg
	100 mg	50 mg	
Titration	Gradual titration every 2–4 weeks based on clinical response and treatment targets		
Excretion	Renal	Hepatic	Hepatic
Notes	Higher potency	Off-label use^‡^	Off-label use^§^
		Higher cost (not government reimbursed in Portugal)	

* In our experience, most patients can achieve therapeutic goals with doses up to 200 mg, and higher doses are rarely required.

^†^ The maximum approved dose by the European Medicines Agency is 50 mg daily.

^‡^ Only approved by the European Medicines Agency for heart failure.

^§^ Only approved by the European Medicines Agency for chronic kidney disease with albuminuria associated with type 2 diabetes.

MRAs, mineralocorticoid receptor antagonists.

Hypokalemia typically improves within days of initiating an MRA. If potassium supplements are being used, they should be gradually reduced within 2–4 days (1). BP responds more gradually, with noticeable effects appearing over 4–8 weeks of therapy, while maximal improvement may take up to 3 months (1, 3). An initial clinical and biochemical evaluation should be performed 1–2 weeks after initiating MRA therapy, assessing BP and serum potassium. A second evaluation should be performed at 4–6 weeks, reassessing BP and potassium and additionally evaluating renal function and plasma renin to guide further dose titration (7, 15). Particular caution is required in patients with CKD, as MRAs may precipitate hyperkalemia or reduce glomerular filtration rate (GFR) (1, 4, 13).

Most authors recommend discontinuing MRAs on postoperative day 1, along with any potassium supplements (2, 4, 7, 13). However, Schreiner *et al.* suggested stopping spironolactone up to 1 week before surgery, based on the long half-life of its active metabolites (19). Currently, evidence is still insufficient to clearly establish the optimal timing for MRA discontinuation. An intermediate approach may therefore be considered, in which the final dose of spironolactone is administered on the morning of the day before surgery, taking into account the 16.5 h half-life of its active metabolite, canrenone (33). This strategy aims to preserve BP control immediately prior to surgery while minimizing circulating active metabolites in the early postoperative period. Whenever feasible, surgery should be scheduled in the morning to align with this pharmacokinetic profile. For patients treated with eplerenone or finerenone, the final dose should typically be administered on the night before surgery, given their shorter half-lives (28, 32).

### Preoperative endocrinology appointment

A preoperative endocrinology appointment is mandatory for all patients with PA who are being considered for adrenalectomy. During this visit, the endocrinologist should review the disease’s physiopathology, discuss the goals and expected outcomes of surgery, and address the expected timeline for postoperative recovery.

Patients must be informed that the full impact of surgery on BP may take 1–3 months and, in some cases, continue to evolve over 6–12 months (3, 4, 7, 13). Moreover, it is essential to disclose that an initial worsening of GFR is expected, reflecting the reversal of chronic aldosterone-mediated glomerular hyperfiltration and unveiling a previous unknown CKD (2, 4, 7, 15). This decline may occur even before surgery, if appropriate MRA therapy is implemented. Approximately 40% of patients may experience a significant reduction in GFR (34), with a median decrease of 10.69 mL/min/1.73 m^2^ reported in a meta-analysis (35). GFR typically stabilizes within 3–6 months postoperatively, with renal function showing better outcomes in the long term (5, 36, 37).

In all cases of adrenal adenoma, cortisol co-secretion must be assessed (1, 5). The reported prevalence of PA with mild-autonomous cortisol secretion (MACS) ranges from 10 to 30% of patients and tends to be higher in larger tumors and advanced age (5, 7, 38). When MACS is present, ACTH-independent cortisol secretion should be confirmed (39). Patients must be informed of possible glucocorticoid insufficiency after surgery, as up to 25–50% may require transient glucocorticoid replacement therapy following adrenalectomy (13, 40). Preoperative ACTH and DHEA-S measurements may help determine the degree of hypothalamic–pituitary–adrenal axis suppression and identify patients at higher risk of adrenal insufficiency (41, 42). Close collaboration with the anesthesiology team is essential, as intraoperative administration of dexamethasone can significantly confound postoperative adrenal function testing and lead to misinterpretation of results.

Finally, considering the adverse myocardial effects of aldosterone excess, including left ventricular hypertrophy and myocardial fibrosis (43), patients should undergo echocardiographic evaluation to detect target organ damage (44). Abnormal findings should be reviewed during clinical visits, as they may reverse with appropriate treatment (2, 45).

## Hospital admission

The hospital admission phase constitutes the second step of the perioperative algorithm and aims to ensure hemodynamic stability, electrolyte control, and appropriate perioperative medication management.

Patients with well-controlled BP and normal potassium levels could be admitted on the day of surgery, an approach facilitated by close coordination with the surgical team. A preoperative endocrinology appointment scheduled shortly before the procedure is important to determine whether earlier hospitalization is needed for further optimization. Upon admission, BP should be monitored at least every 6 h, serum potassium should be rechecked if recent measurements (within the previous 2–4 weeks) are unavailable, and usual antihypertensive therapy should be continued, with the exception of MRAs.

For patients with cortisol co-secreting adenomas, the optimal timing of perioperative glucocorticoid administration remains a matter of debate, with recommendations for both intraoperative and postoperative initiation (5, 13). One commonly adopted approach is to provide intraoperative glucocorticoid coverage using a single 100 mg dose of hydrocortisone, with the need for additional supplementation reassessed on postoperative day one, as discussed below (41, 46, 47).

## Postoperative management

The postoperative phase represents the final step of the perioperative algorithm and focuses on early detection of complications, safe antihypertensive down-titration, and assessment of biochemical and clinical outcomes.

In the postoperative period, the main complication of concern is hyperkalemia due to transient hyporeninemic hypoaldosteronism, resulting from the prolonged suppression of the renin–angiotensin–aldosterone axis and the reduced function of the contralateral adrenal gland. Hyperkalemia occurs in approximately 5–10% of cases (7, 48). Several risk factors have been identified, including reduced preoperative GFR, postoperative increases in creatinine, adenomas larger than 2 cm, older age, and long-standing HT (3, 4, 5, 7). Most cases are mild and transient, and only around 5% of patients require mineralocorticoid replacement therapy (4). The preoperative use of MRAs, particularly when titrated to raise renin levels, may reduce the likelihood of this complication by improving contralateral adrenal responsiveness (49). A reduction in GFR is also expected postoperatively, as previously mentioned.

On the first postoperative day, fasting measurements of aldosterone, potassium, and renal function should be obtained between 08:00 and 09:00 h. In cases of cortisol co-secretion, morning cortisol should also be assessed (4, 7). BP should continue to be monitored every 6 h, and antihypertensive therapy is reduced by approximately half, both in the number of agents and in dosages (3, 7). MRAs should not be resumed, and medications that increase the risk of hyperkalemia, such as angiotensin-converting enzyme inhibitors or angiotensin receptor blockers, should be avoided (7). Potassium supplementation should be discontinued unless the serum potassium level is below 3 mmol/L (4). A high-sodium diet is recommended to expand intravascular volume, increase distal sodium delivery to the distal nephron, and thereby promote potassium kidney excretion (4, 5, 7, 13). When intravenous fluids are required, potassium-free 0.9% sodium chloride solution is preferred (4, 7).

Aldosterone levels measured on postoperative day 1 are not sufficient to establish surgical cure but may still provide clinically relevant information (50). William F Young suggested that patients with aldosterone concentrations >5 ng/dL should be closely monitored for potential persistence of PA (36). However, in patients who receive preoperative MRA therapy leading to loss of renin suppression, aldosterone levels may exceed 5 ng/dL despite eventual biochemical cure, likely reflecting early recovery of contralateral adrenal function. Ishihara *et al.* (51) identified an optimal cutoff of 8.1 ng/dL for early postoperative aldosterone assessment (within 10 days after surgery), with a sensitivity of 76.5% and specificity of 100%. In that cohort, 51 of 59 patients had received preoperative MRA therapy. Nevertheless, median preoperative PRA remained <1 ng/mL/h, raising concerns about the applicability of this threshold in patients treated with MRAs who do not exhibit suppressed renin preoperatively. Overall, evidence remains limited to define robust early postoperative aldosterone cutoff values to guide clinical decision-making. In cases of elevated aldosterone levels, particularly when associated with persistent hypokalemia or lack of BP improvement, suspicion for persistent PA should be raised, and repeat biochemical evaluation after the recommended 4-week MRA washout is warranted (13).

In patients with cortisol co-secretion, evaluation for postoperative glucocorticoid insufficiency usually relies on the morning cortisol level obtained on postoperative day 1, in the absence of intraoperative dexamethasone administration (1). Cortisol levels below 5 μg/dL are strongly suggestive of adrenal insufficiency and warrant initiation of hydrocortisone therapy. However, reported cortisol thresholds to exclude adrenal insufficiency vary, and no universal consensus has been established (46). Levels above 14.5 μg/dL are generally considered indicative of adequate adrenal reserve (52). For intermediate cortisol levels (between 5 and 14.5 μg/dL), adrenal insufficiency cannot be reliably confirmed or excluded. Therefore, empirical hydrocortisone therapy should be considered. Clinical judgment remains essential, particularly in patients with preoperative evidence of hypothalamic–pituitary–adrenal axis suppression, such as low ACTH or DHEA-S levels (41). If the patient remains hospitalized, repeat assessment on postoperative day 2 may help clarify adrenal function and guide further management. In cases of diagnostic uncertainty, a corticotropin stimulation test may be performed, as it remains the gold standard for the diagnosis of adrenal insufficiency (46). In the presence of adrenal insufficiency, perioperative glucocorticoid replacement may be initiated as a continuous infusion of hydrocortisone 200 mg over 24 h or, if continuous infusion is not feasible, as intermittent boluses of 50 mg every 6 h (46). In patients with a favorable clinical course and once oral intake is established, therapy may be transitioned to oral hydrocortisone 40 mg daily in three divided doses using a circadian regimen. Subsequent tapering should be guided by clinical symptoms and biochemical assessments (53).

### Discharge

Young patients with well-controlled BP, preserved renal function, and normal potassium levels at the first postoperative evaluation may be discharged the same day, provided that close outpatient follow-up is feasible.

Patients with cortisol co-secretion should be educated on the symptoms of adrenal insufficiency and emergency management (46). In selected cases, a standby hydrocortisone prescription may be provided for use as needed. All patients should be advised to maintain a non-restricted sodium diet and to perform daily home BP monitoring (7, 48).

### Follow-up

During the first 4 weeks after surgery, weekly evaluations are recommended to monitor potassium levels, review medications, and assess BP (7). This schedule is supported by evidence indicating that postoperative hyperkalemia can develop in this period (54). At approximately 3 months postoperatively, patients undergo reassessment of BP, potassium levels, and medication requirements (6). Renal function is also re-evaluated, as GFR typically stabilizes by this time (37).

The final assessment of surgical outcomes is performed at 6–12 months after adrenalectomy, in accordance with the primary aldosteronism surgical outcome (PASO) consensus (6). Surgical outcomes are defined based on the evaluation of both clinical and biochemical responses, each classified as complete, partial, or absent (Table 2). Clinical outcomes are determined by comparing pre- and postoperative BP values and antihypertensive medication requirements. According to the PASO consensus, a significant change in BP is defined as a difference of at least 20 mmHg in systolic or 10 mmHg in diastolic values, in either direction. Changes in antihypertensive therapy are quantified using the anatomical therapeutic chemical/defined daily dose (ATC/DDD) index (55) and are considered significant if the total daily dose differs by at least 0.5, in either direction. Biochemical outcomes are assessed by measuring aldosterone, renin, and potassium levels.

**Table 2:** Biochemical and clinical responses defined by the PASO consensus.

	Biochemical response	Clinical response
Complete	Correction of hypokalemia, if present, and normalization of aldosterone/renin ratio or aldosterone suppression in confirmatory test^*^	Normal BP^†^ without AHTM
Partial	Correction of hypokalemia, if present, with aldosterone/renin ratio increased but with ≥50% decrease in baseline aldosterone or improved confirmatory test result	Same BP with less AHTM or reduction in BP with same or less AHTM
Absent	Persistent hypokalemia, if present, and/or persistent raised aldosterone/renin ratio, with failure to suppress aldosterone in confirmatory test	Same or increased BP with same or increased AHTM


AHTM, antihypertensive medication; BP, blood pressure.

* If aldosterone/renin ratio is increased.

^†^ As defined by the European Society of Hypertension Guidelines (58).

### Histological analysis

From a histopathological standpoint, the introduction of the International Histopathology Consensus Classification for Unilateral Primary Aldosteronism has underscored the prognostic value of specific histological patterns in predicting disease recurrence (56). Classic histology is defined by the presence of a single aldosterone-producing adenoma or nodule, whereas non-classic histology encompasses multiple nodules, micronodules, or diffuse hyperplasia. Patients with non-classical findings appear to have a higher risk of recurrence and therefore warrant closer follow-up (13).

More recently, an adjunct to CYP11B2 immunohistochemistry has been proposed to further refine this classification. In cases where more than one nodule is identified, the authors introduce the ‘B2 ratio’, defined as the diameter of the largest CYP11B2-positive nodule divided by that of the second largest. A B2 ratio ≥8.1 supports classification as classic histology, as the smaller nodule likely represents background ‘noise’ rather than an independent source of aldosterone excess (57).

### Discharge of the endocrinology department

Patients who achieve complete biochemical success along with complete or partial clinical response and have classic histopathological findings can be discharged from specialized endocrinology follow-up and transitioned to long-term care with their general practitioners. Annual assessments, including BP and serum potassium measurements, are recommended (6). If recurrence of PA is suspected, aldosterone, renin, and potassium should be re-evaluated, and any abnormalities should prompt re-referral to endocrinology for further investigation.

## Conclusion

A structured perioperative approach is essential for optimizing outcomes in patients with unilateral PA. The proposed perioperative algorithm provides a practical and reproducible framework for managing patients undergoing adrenalectomy. Timely MRA initiation, comprehensive preoperative assessment, and vigilant postoperative monitoring help minimize complications and support safe recovery. Accurate and standardized evaluation of surgical outcomes allows meaningful comparison across centers, while integration of histopathological findings guides long-term management and facilitates an appropriate transition back to primary care setting.

## Declaration of interest

The authors declare that there is no conflict of interest that could be perceived as prejudicing the impartiality of the work reported.

## Funding

This review did not receive any specific grant from any funding agency in the public, commercial, or not-for-profit sector.

## Author contribution statement

All authors contributed to the statements presented in this review. PB wrote the initial draft of the manuscript. All authors reviewed and approved the manuscript.
